# Detection of Alterations in the Gut Microbiota and Intestinal Permeability in Patients With Hashimoto Thyroiditis

**DOI:** 10.3389/fimmu.2021.579140

**Published:** 2021-03-05

**Authors:** Leonardo César de Freitas Cayres, Larissa Vedovato Vilela de Salis, Guilherme Siqueira Pardo Rodrigues, André van Helvoort Lengert, Ana Paula Custódio Biondi, Larissa Donadel Barreto Sargentini, João Luiz Brisotti, Eleni Gomes, Gislane Lelis Vilela de Oliveira

**Affiliations:** ^1^Microbiome Study Group, School of Health Sciences Dr. Paulo Prata, São Paulo, Brazil; ^2^Microbiology Program, Institute of Biosciences, Humanities and Exact Sciences (IBILCE), São Paulo State University (UNESP), São Paulo, Brazil; ^3^Barretos Cancer Hospital, São Paulo, Brazil; ^4^Food Engineering and Technology Department, Institute of Biosciences, Humanities and Exact Sciences, São Paulo State University (UNESP), São José do Rio Preto, Brazil

**Keywords:** intestinal dysbiosis, inflammatory cytokines, dietary habits, gut microbiota, Hashimoto thyroiditis, autoimmune disease, intestinal permeability

## Abstract

Hashimoto thyroiditis (HT) is the most common autoimmune disease worldwide, characterized by chronic inflammation and circulating autoantibodies against thyroid peroxidase and thyroglobulin. Patients require hormone replacement with oral levothyroxine, and if untreated, they can develop serious adverse health effects and ultimately death. There is a lot of evidence that the intestinal dysbiosis, bacterial overgrowth, and increased intestinal permeability favor the HT development, and a thyroid–gut axis has been proposed, which seems to impact our entire metabolism. Here, we evaluated alterations in the gut microbiota in Brazilian patients with HT and correlated this data with dietary habits, clinical data, and systemic cytokines and zonulin concentrations. Stool samples from 40 patients with HT and 53 controls were analyzed using real-time PCR, the serum cytokine levels were evaluated by flow cytometry, zonulin concentrations by ELISA, and the dietary habits were recorded by a food frequency questionnaire. We observed a significant increase (*p* < 0.05) in the *Bacteroides* species and a decrease in *Bifidobacterium* in samples of patients with HT. In addition, *Lactobacillus* species were higher in patients without thyroid hormone replacement, compared with those who use oral levothyroxine. Regarding dietary habits, we demonstrated that there are significant differences in the consumption of vegetables, fruits, animal-derived proteins, dairy products, saturated fats, and carbohydrates between patients and control group, and an inverse correlation between animal-derived protein and *Bacteroides* genus was detected. The microbiota modulation by diet directly influences the inflammatory profile due to the generated microbiota metabolites and their direct or indirect action on immune cells in the gut mucosa. Although there are no differences in systemic cytokines in our patients with HT, we detected increased zonulin concentrations, suggesting a leaky gut in patients with HT. These findings could help understand the development and progression of HT, while further investigations to clarify the underlying mechanisms of the diet–microbiota–immune system axis are still needed.

## Introduction

The Hashimoto thyroiditis (HT) is an organ-specific autoimmune disease and one of the most common diseases worldwide (1). HT is characterized by chronic inflammation, with tertiary lymphoid follicles development and increased concentrations of circulating autoantibodies against thyroid peroxidase (anti-TPO) and thyroglobulin (anti-Tg) (2). The thyroid parenchyma is replaced by the lymphocytes infiltration, inducing organ enlargement, gland fibrosis, and destruction (3). The progressive thyrocyte depletion leads to decreased thyroid hormone function and clinical hypothyroidism, a condition marked by a reduced metabolic activity in several tissues. The disease is correlated with decreased cardiovascular contractility, coronary artery disease, infertility, dementia, neurosensory and musculoskeletal changes, and reduced colonic activity and orocecal time transit (4, 5). Patients require hormone replacement with oral levothyroxine, and if untreated, they can develop serious adverse health effects and ultimately death (6, 7).

Hashimoto thyroiditis has become a global public health concern, and the worldwide prevalence reaches 10–12%, affecting 10 times more women, and the peak incidence is between 30 and 50 years old (5, 6). In the United States of America, HT affects ~4% of women aged 18–24 years and 21% of women older than 74 years (6). In Europe, the prevalence varies between 0.2 and 5.3% in the general population, and 7.5% of women in the United Kingdom presented elevated thyroid-stimulating hormone (TSH) concentrations (8). In Brazil, the prevalence of elevated TSH in adult women in Rio de Janeiro was 12.3%, reaching 19.1% among those over 70 years old. In the metropolitan region of São Paulo, the HT prevalence reached 8.0%, and in an elderly population, the prevalence was 6.5 and 6.1% for women and men, respectively (9). The economic impact can be significant taking into account the direct effects of the disease, with the HT side effects, which range from depression and to a greater propensity to develop differentiated thyroid cancer and extra-thyroidal cancers (5, 10–13).

Although the HT etiology remains unknown, epidemiological studies suggest that HT is caused by an interaction between genetic and environmental factors (14). The genetic predisposition plays a crucial role in the loss of tolerance to self-antigens and *loci* linked to immune-modifying genes such as human leukocyte antigens (HLA class I and II) and cytotoxic-T-lymphocyte-associated protein 4 (CTLA-4) could be involved in the autoimmune process. The interactions between these *loci* and environmental factors might influence disease phenotype and severity (3). The environmental factors that may be involved in the HT triggering include excessive iodine consumption; selenium, iron, zinc, and vitamin D deficiencies; bacterial infections, such as *Helicobacter pylori* and *Yersinia enterocolitica*; viral infections, such as hepatitis C, Epstein–Barr virus, and enteroviruses; smoking; pollutants; and chemical agents, and also, factors such as stress, climate, age, and gender (15). More recently, it is hypothesized that gut microbiota might play an important role in triggering HT (16).

There is a lot of evidence that the intestinal dysbiosis, bacterial overgrowth, and increased intestinal permeability (leaky gut) favor HT development, and a thyroid–gut axis has been proposed which seems to impact our entire metabolism (17, 18). The commensal gut bacteria influence the innate, cellular, and humoral immunity by interacting with epithelial cells and mucosal immune cells *via* pattern recognition receptors. Depending on the generated metabolites, microbiota can activate a proinflammatory or anti-inflammatory program (19). Moreover, gut microbiota can affect the thyroid hormone concentrations by controlling iodine uptake, degradation, and enterohepatic recycling of these hormones, and the levothyroxine bioavailability (L-thyroxine). There is a prominent effect of minerals on microbiota–host interactions, mainly by zinc, selenium, and iron. In addition, the microbiota may play role in thyroid disorders by influencing neurotransmitters, the hypothalamus–pituitary axis, the dopamine production, and consequently the TSH secretion (17).

The short-chain fatty acids (SCFAs), metabolites from anaerobic microbiota fermentation, function as an energy source for enterocytes and, together with thyroid hormones, mainly T3, induce enterocyte differentiation, and strengthen the epithelial barrier integrity (20). In addition to SCFAs, the microbiota produce secondary bile acids in the colon, which present systemic effects and interfere with TSH levels (21). Secondary bile acids can regulate type 2 iodothyronine deiodinase (D2) in the gut, molecules involved in thyroid metabolism, and the lipopolysaccharides (LPS) derived from Gram-negative bacteria inhibits intestinal D2 and hepatic D1, and decreases the expression of thyroid hormones in the liver (22).

In animal models, the association between the gut microbiota and thyroid functions has been proposed since 1970s. The depleted microbiota in antibiotic-treated rats induced a decrease in thyroid functions, when evaluated by radioactive iodine uptake (23). In the same line, the germ-free mice present an increase in TSH levels when compared with the conventionally raised mice (22).

In humans, few studies have been carried out in order to understand the relationship between intestinal microbiota and HT. Some recent studies have demonstrated the existence of intestinal dysbiosis in patients with HT and showed an increase in the *Bacteroides, Escherichia-Shigella*, and *Parasutterella* genera and a decrease in the *Bifidobacterium, Lactobacillus, Prevotella*, and *Dialister* genera in the gut microbiota in patients with HT (23). Another study showed an increase in the relative abundance of *Blautia, Roseburia, Ruminococcus, Romboutsia, Dorea, Fusicatenibacter*, and *Eubacterium* genera and a decrease in *Bacteroides, Faecalibacterium, Prevotella*, and *Lachnoclostridium* genera in the gut microbiota in patients with HT (24).

On the basis of this background and the fact that there are no studies evaluating the gut microbiota in Brazilian patients with HT, the aim of this study was to evaluate the presence of some specific bacteria in stool samples of patients with HT and to correlate these data with dietary habits, clinical data, and systemic cytokines and zonulin concentrations.

## Materials and Methods

### Patients With HT and Control Subjects

Patients diagnosed with HT, with increased TSH and decreased free thyroxine levels (FT4), associated or not associated with elevated anti-TPO and anti-Tg antibodies, were enrolled for this study. The Barretos Cancer Hospital Ethics Committee (Process number 1,359/2017) approved this study, and all participants signed the informed consent form.

About 40 patients with HT aged 23–82 years (mean age ± SD = 48.9 ± 13.3 years) were enrolled. Fifty-three healthy controls aged 18–79 years (mean age ± SD = 45.6 ± 16.7 years) were recruited for the study. Exclusion criteria, for both groups, include the use of anti-inflammatories, immunosuppressant drugs, antibiotics, and vaccination in the last 30 days, with gastrointestinal surgeries, inflammatory bowel diseases, and chronic diarrheas.

After the informed consent, patients and controls answered a food frequency questionnaire (FFQ), containing questions about frequency and consumption of vegetables, fruits, animal-derived proteins, dairy products, carbohydrates, and saturated fat. After that, the peripheral blood was collected, and stool samples were collected within 3–5 days. Table 1 summarizes demographic data and clinical parameters in patients with HT.

**Table 1:** Demographic data and clinical parameters from patients with Hashimoto thyroiditis (HT).

**Patients**	**Gender/Age**	**Ethnicity**	**TSH (μUI/ml)**	**FT4 (ng/dl)**	**TPO Ab (UI/ml)**	**Tg Ab (UI/ml)**	**Disease duration (years)**	**Current treatment**
HT01	F/28	Caucasian	ND	ND	ND	ND	08 y	LT4
HT02	F/57	Caucasian	1.61	0.84	51.27	ND	01 y	NR
HT03	F/35	Caucasian	ND	ND	ND	ND	01 y	NR
HT04	F/28	Afrodescendent	ND	ND	ND	ND	05 y	NR
HT05	F/55	Caucasian	ND	ND	ND	ND	03 y	LT4
HT06	F/29	Caucasian	0.01	2.6	356	ND	13 y	NR
HT07	F/56	Caucasian	5.17	1.23	ND	ND	25 y	LT4
HT08	F/26	Caucasian	6.55	1.2	>600	241.3	04 y	LT4
HT09	F/42	Caucasian	9.86	ND	71	ND	12 y	LT4
HT10	F/67	Caucasian	12.21	1.1	463	ND	05 y	LT4
HT11	F/40	Caucasian	17	0.92	325	ND	03 y	LT4
HT12	M/58	Caucasian	7.58	1.2	ND	ND	05 y	LT4
HT13	F/50	Afrodescendent	51	0.3	109	111	02 y	LT4
HT14	F/53	Afrodescendent	14	0.87	1,683	6,652	42 y	LT4
HT15	F/54	Caucasian	3.41	1.21	ND	ND	25 y	NR
HT16	F/64	Caucasian	1.53	1.84	>2,000	ND	25 y	LT4
HT17	F/52	Caucasian	9.85	0.57	250	11.58	05 y	LT4
HT18	F/45	Caucasian	5.51	0.62	160	137.8	02 y	LT4
HT19	F/63	Afrodescendent	ND	ND	ND	ND	17 y	LT4
HT20	F/53	Caucasian	ND	ND	ND	ND	10 y	LT4
HT21	F/82	Afrodescendent	ND	ND	ND	ND	10 y	NR
HT22	F/54	Caucasian	ND	ND	211.8	56.53	20 y	LT4
HT23	F/38	Afrodescendent	ND	ND	ND	ND	14 y	LT4
HT24	F/65	Caucasian	4.52	ND	ND	ND	10 y	LT4
HT25	F/52	Afrodescendent	10.1	ND	ND	ND	06 y	LT4
HT26	F/65	Afrodescendent	4.72	ND	ND	ND	05 y	LT4
HT27	M/62	Caucasian	16.8	2.05	10.82	42.81	03 y	LT4
HT28	F/47	Caucasian	1.26	1.1	ND	ND	21 y	LT4
HT29	F/35	Caucasian	9.48	ND	ND	ND	07 y	LT4
HT30	F/51	Afrodescendent	3.2	0.89	270	143.23	18 y	NR
HT31	F/45	Caucasian	4.33	1.06	159	214	04 y	LT4
HT32	M/66	Caucasian	ND	ND	ND	ND	03 y	NR
HT33	M/56	Caucasian	0.52	1.73	970	26	0.9 y	LT4
HT34	F/23	Caucasian	2.03	0.96	1,840	8.58	05 y	LT4
HT35	F/44	Caucasian	0.97	ND	ND	ND	07 y	LT4
HT36	F/45	Caucasian	ND	ND	ND	ND	ND	LT4
HT37	F/49	Caucasian	4.91	0.95	0.52	57	07 y	LT4
HT38	F/43	Caucasian	0.48	1.1	366	5.06	02 y	LT4
HT39	F/52	Caucasian	4.89	1.24	282	>1,000	10 y	LT4
HT40	F/27	Caucasian	ND	ND	ND	ND	ND	LT4

*HT, Hashimoto thyroiditis; F, female; M, male; TSH, thyroid-stimulating hormone (Reference: 0.38–5.33 μUI/ml); FT4, free thyroxine (Reference: 0.54–1.24 ng/dl); TPO Ab, anti-thyroid peroxidase antibodies (Reference: <9 UI/ml); Tg Ab, anti-thyroglobulin antibodies (Reference: <4 UI/ml); y, years; ND, not determined; LT4, oral levothyroxine sodium; NR, non-thyroid hormone replacement*.

### DNA Extraction From Stool Samples and Real-Time PCR

DNA was obtained from 200 mg of stool samples of patients with HT and controls by using QIAamp Fast DNA Stool Mini Kit (QIAGEN Inc., Hilden, Germany), according to the instructions of the manufacturer. The composition of the gut microbiota was determined by specific primers for *Bacteroides* (*Bac*), *Bifidobacterium* (*Bif*), *Clostridium coccoides* (*Ccoc*), *Clostridium coccoides–Eubacterium rectale* (*CIEub*), *Clostridium leptum* (*Clept*), *Lactobacillus* (*Lac*), *Prevotella* (*Prev*), and *Roseburia* (*Ros*). The primers were described previously and designed by using the 16S rRNA gene sequences from the Ribosomal Database Project (25). PCR was performed by using 7.5 μl of Power SYBR Green PCR Master Mix (Applied Biosystems, Life Technologies, Carlsbad, CA, USA), 4.0 μl of ultrapure water (Uniscience Corporation, Hialeah, FL, USA), 1.0 μl of each primer (2 μM), and 1.5 μl of DNA (5 ng). The normalization was performed by dividing the DNA copy numbers obtained for target primers (*Bac, Bif, Ccoc, CIEub, Clept, Lac, Prev*, and *Ros*) and for the copy numbers of universal primer (*Univ*). The relative abundance was quantified by using cycle threshold (Ct) values and was expressed in relative expression units (REUs) per 200 mg of stool (26).

### Cytokine Serum Quantification by Flow Cytometer

Approximately 8.5 ml of peripheral blood were collected from patients with HT and healthy individuals in gel tube with clot activator (BD Vacutainer SST II Advance, BD Biosciences, Franklin Lakes, NJ, USA). After collection, samples were incubated at room temperature for 1 h and centrifuged at 1.372 × g for 5 min. The supernatant was withdrawn, and the serum was used for cytokine quantification that was performed by cytometric bead array assay (Human Th1/Th2/Th17 Kit, BD Biosciences, San Jose, CA, USA). The levels of interleukin (IL)-2, IL-4, IL-6, IL-10, IL-17A, interferon-gamma (IFN-γ), and tumor necrosis factor (TNF) were detected by a flow cytometer (FACSCanto™ II, BD Biosciences, Franklin Lakes, NJ, USA). Results were analyzed by BDFCAP array™ software and were expressed in pg/ml.

### Zonulin Serum Quantification by Sandwich-ELISA

After peripheral blood collection, samples were incubated in gel tube with clot activator for 50 min and then centrifuged at 1.372 × g for 5 min. The serum samples were used for zonulin quantification by using the Human Zonulin ELISA Kit (Elabscience, Bethesda, MD, USA). Standards and samples were added to plates precoated with specific antibodies to human zonulin and incubated for 1 h at 37°C. Then, a biotinylated detection antibody specific for human zonulin and avidin–horseradish peroxidase conjugate was added and incubated for 30 min. Unbound and free molecules were washed away. The substrate solution was added to each well and incubated for 15 min. When the enzyme–substrate reaction was blocked by stop solution, the color turned to yellow. The optical density was measured in a spectrophotometer at 450 nm. The standard curve was constructed, and the zonulin concentrations were calculated by converting the obtained optical density in ng/ml.

### Statistical Analysis

The data extracted from the surveys on dietary habits of patients with HT and controls were analyzed by the Pearson chi-square test, the REUs of the gut microbiota were analyzed by the non-parametric Mann–Whitney *U*-test, and the serum concentrations of cytokines and zonulin were performed by unpaired *t*-test with the Welch's correction. Correlations of the gut microbiota, clinical data, cytokines, and zonulin concentrations were performed by the Spearman's rank correlation coefficient. Values of *p* < 0.05 were considered statistically significant.

## Results

### Increased *Bacteroide*s and Decreased *Bifidobacterium* in the Gut Microbiota in Patients

**Figure 1:**
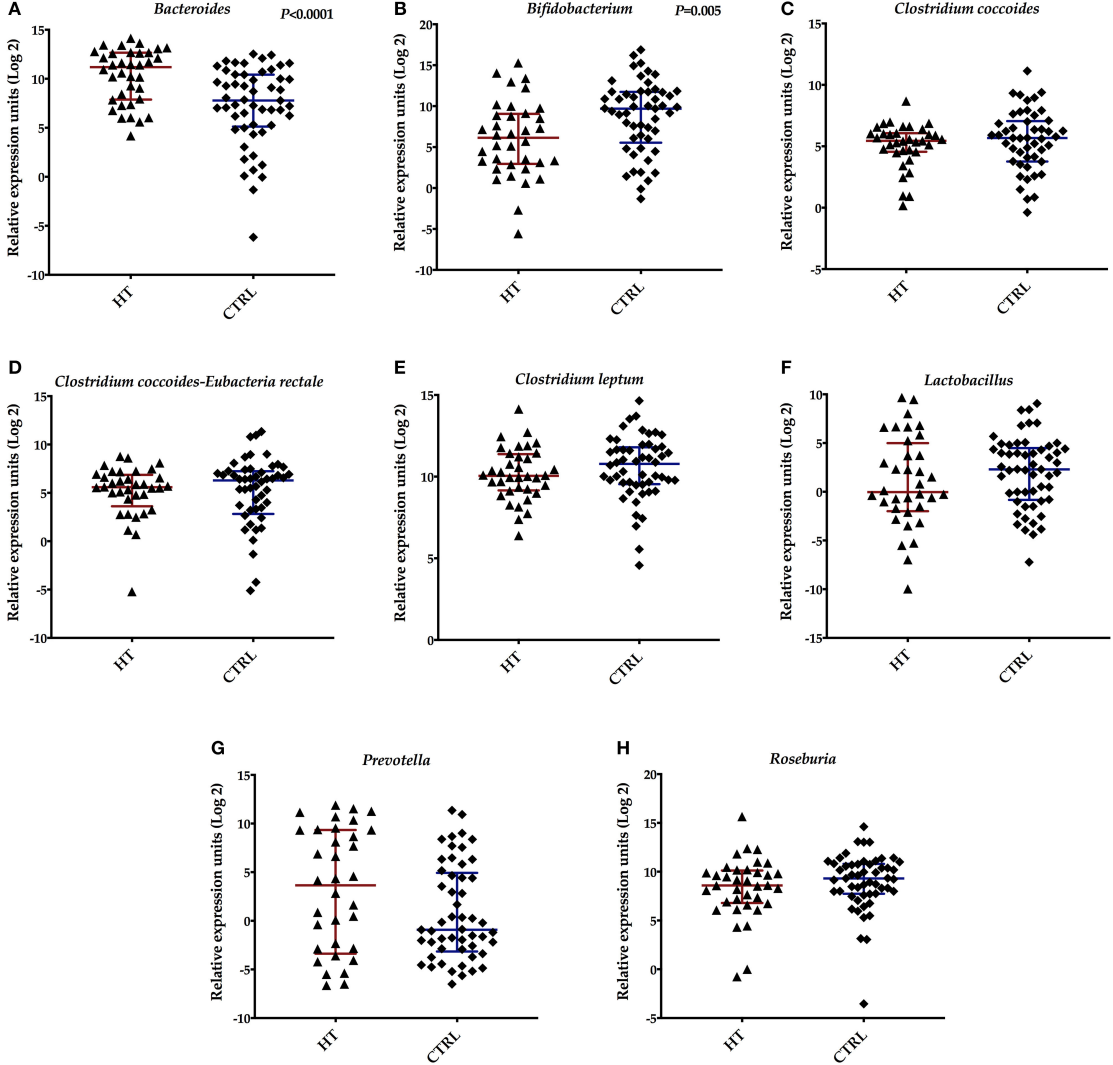
Relative expression units (REU) of the intestinal microbiota found in stool samples from patients with Hashimoto thyroiditis (HT) and healthy controls (CTRL). **(A)** *Bacteroides*, **(B)** *Bifidobacterium*, **(C)** *Clostridium coccoides*, **(D)** *Clostridium coccoides-Eubacterium-rectale*, **(E)** *Clostridium leptum*, **(F)** *Lactobacillus*, **(G)** *Prevotella*, and **(H)** *Roseburia* species. Bars represent the median with interquartile range of REU per 200 mg of stool.

To analyze the composition of the gut microbiota in patients with HT, we investigated some bacterial species in stool samples by real-time PCR. We detected an increase in the REUs of *Bacteroides* species in the samples derived from patients (median *Bac*: 2,344 REU/200 mg stool; *p* < 0.0001) compared with control subjects (median *Bac*: 221.3 REU/200 mg stool) (Figure 1A). A significant decrease in the REUs of *Bifidobacterium* species was observed in the stool samples isolated from patients with HT (median *Bif* : 70.86 REU/200 mg stool; *p* = 0.005) when compared with controls (median *Bif* : 830.6 REU/200 mg stool) (Figure 1B). There are no significant differences (*p* > 0.05) in the REU/200 mg stool obtained from *Clostridium coccoides* (median *Ccoc*: 43.51), *Clostridium coccoides-Eubacteria rectale* (median *CIEub*: 48.03), *Clostridium leptum* (median *Clept*: 1.060), *Lactobacillus* (median *Lac*: 0.974), *Prevotella* (median *Prev*: 12.53), and *Roseburia* species (median: 385.8) in stool samples of patients with HT, when compared with the healthy individuals (median *Ccoc*: 51.21; *CIEub*: 78.04; *Clept*: 1,762; *Lac*: 4.933; *Prev*: 0.530; and *Ros*: 635.9, respectively) (Figures 1C–H).

### Dietary Habits and Correlations With the Gut Microbiota

To evaluate the dietary habits from patients and controls, we applied an FFQ regarding the frequency of consumption of vegetables, fruits, animal-derived proteins, dairy products, saturated fats, and carbohydrates. The interviewed subjects reported the daily ingestion of vegetables [patients (HT) = 59%; controls (C) = 53.3%; fruits (HT = 38.5%; C = 43.3%), proteins (HT = 51.3%; C = 30%), dairy products (HT = 48.7%; C = 60%), saturated fats (HT = 17.9%; C = 16.7%), and carbohydrates (HT = 38.5%; C = 43.3%)]. When we compared the dietary habits between patients with HT and controls, we observed a significant difference (*p* < 0.05) in the consumption of vegetables, fruits, proteins, dairy products, saturated fats, and carbohydrates. The results concerning the dietary habits from patients and controls were shown in Table 2.

**Table 2:** Dietary habits of the patients with HT and control subjects.

**Dietary variables (consumption frequency)**	**Patients with HT *N* (%)**	**Controls *N* (%)**	***p-*value**
**VEGETABLES**			
Less than once per month	1 (2.6%)	0	*p* < 0.001
1–3 times per month	2 (5.1%)	2 (6.7%)	
1–2 times a week	2 (5.1%)	4 (13.3%)	
On most days	11 (28.2%)	8 (26.7%)	
Every day	23 (59%)	16 (53.3%)	
**FRUITS**			
Less than once per month	3 (7.7%)	0	*p* < 0.001
1–3 times per month	4 (10.3%)	4 (13.3%)	
1–2 times a week	7 (17.9%)	8 (26.7%)	
On most days	10 (25.6%)	5 (16.7%)	
Every day	15 (38.5%)	13 (43.3%)	
**PROTEINS**			
1–3 times per month	1 (2.6%)	1 (3.3%)	*p* < 0.001
1–2 times a week	6 (15.4%)	11 (36.7%)	
On most days	12 (30.8%)	9 (30.0%)	
Every day	20 (51.3%)	9 (30.0%)	
**DAIRY PRODUCTS**			
Never consumes	5 (12.8%)	1 (3.3%)	*p* < 0.001
Less than once per month	3 (7.7%)	0	
1–3 times per month	4 (10.3%)	3 (10.0%)	
1–2 times a week	3 (7.7%)	6 (20.0%)	
On most days	5 (12.8%)	2 (6.7%)	
Every day	19 (48.7%)	18 (60.0%)	
**SATURATED FAT**			
Never consumes	7 (17.9%)	7 (23.3%)	*p* = 0.005
Less than once per month	2 (5.1%)	1 (3.3%)	
1–3 times per month	8 (20.5%)	10 (33.3%)	
1–2 times a week	11 (28.2%)	6 (20.0%)	
On most days	4 (10.3%)	1 (3.3%)	
Every day	7 (17.9%)	5 (16.7%)	
**CARBOHYDRATES**			
Never consumes	1 (2.6%)	0	*p* < 0.001
Less than once per month	2 (5.1%)	0	
1–3 times per month	6 (15.4%)	2 (6.7%)	
1–2 times a week	13 (33.3%)	6 (20.0%)	
On most days	2 (5.1%)	9 (30.0%)	
Every day	15 (38.5%)	13 (43.3%)	

In order to detect correlations between diet and gut microbiota composition in patients with HT, we used the consumption frequencies and the REUs of *Bacteroides, Bifidobacterium, Clostridium coccoides, Clostridium coccoides-Eubacteria rectale, Clostridium leptum, Lactobacillus, Prevotella*, and *Roseburia*. We found an inverse correlation (*p* = 0.002; *r* = −0.61) between animal-derived protein consumption by patients and the REUs of *Bacteroides* species.

### Correlations Between the Gut Microbiota and Clinical Data

**Figure 2:**
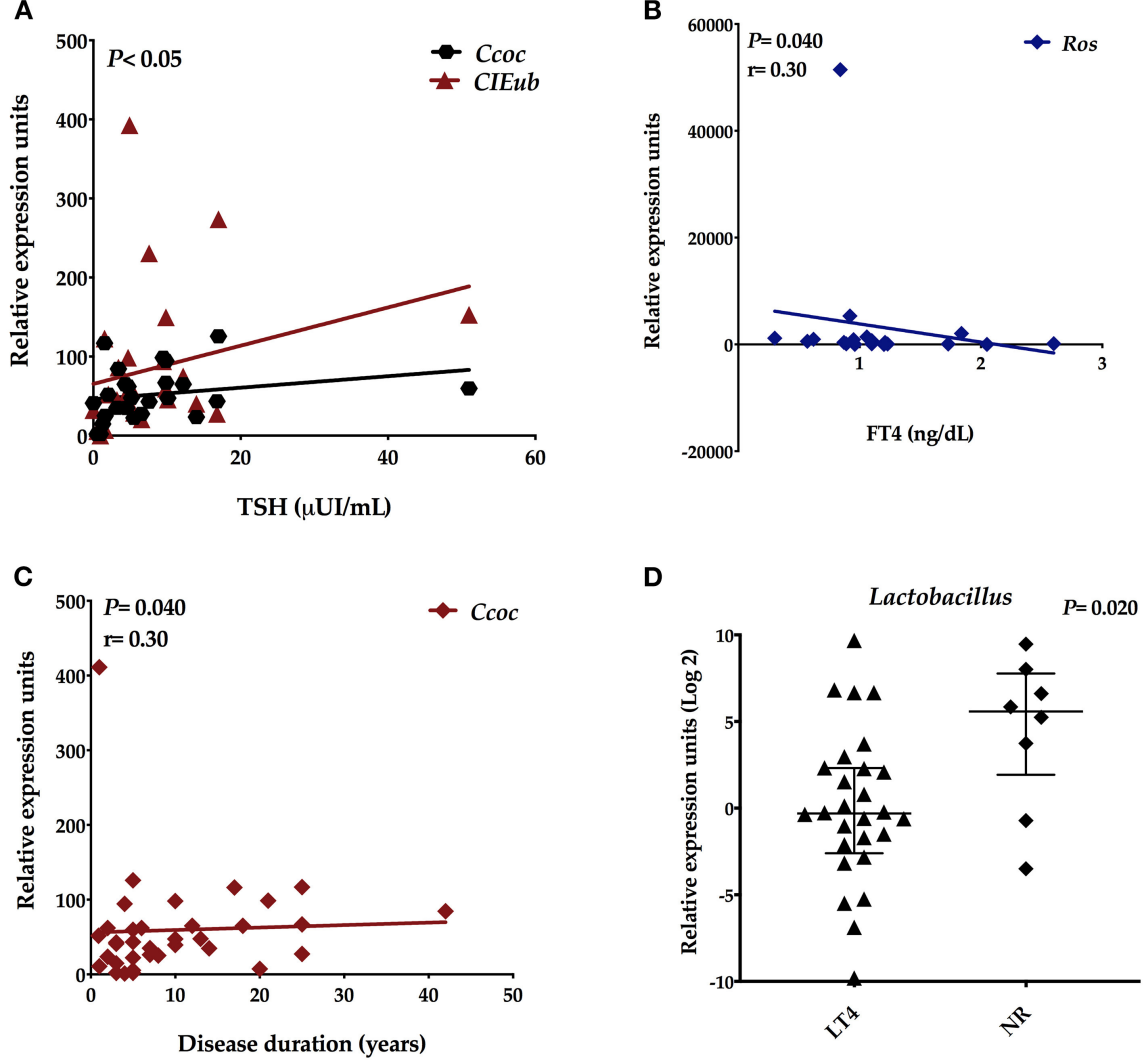
Spearmans' correlation between the relative expression units (REU) of the intestinal microbiota and clinical data. **(A)** REU of *Clostridium coccoides* and *Clostridium coccoides*-Eubacterium-rectale with TSH concentrations, **(B)** REU of Roseburia with FT4 levels, **(C)** REU of *Clostridium coccoides* with disease duration, and **(D)** REU of *Lactobacillus* species in treated patients (LT4) and patients with no hormone replacement (NR).

To evaluate the connection between gut microbiota composition and clinical parameters, we correlated the REUs of *Bacteroides, Bifidobacterium, Clostridium coccoides, Clostridium coccoides-Eubacteria rectale, Clostridium leptum, Lactobacillus, Prevotella*, and *Roseburia* with concentrations of TSH, FT4, TPO, and Tg autoantibodies. We detected positive correlations among the REUs of *Clostridium coccoides* (*p* = 0.023; *r* = 0.40) and *Clostridium coccoides–Eubacteria rectale* (*p* = 0.010; *r* = 0.45) and in the TSH levels (Figure 2A). The REUs of *Roseburia* species inversely correlated (*p* = 0.015; *r* = −0.49) with FT4 levels (Figure 2B). We also found a positive correlation between *Clostridium coccoides* (*p* = 0.040; *r* = 0.30) and disease duration (Figure 2C). There are no correlations (*p* > 0.05) among gut microbes and TPO and Tg autoantibodies. Besides that, we observed significant differences in the REUs of *Lactobacillus* between patients who did thyroid hormone replacement therapy (*N* = 28) and those who did not (*N* = 8) (median *Lac* LT4: 0.8065 REU/200 mg stool; median *Lac* NR: 47.73; *p* = 0.020; Figure 2D).

### Systemic Cytokine Profile in Patients With HT Was Similar to Controls

**Figure 3:**
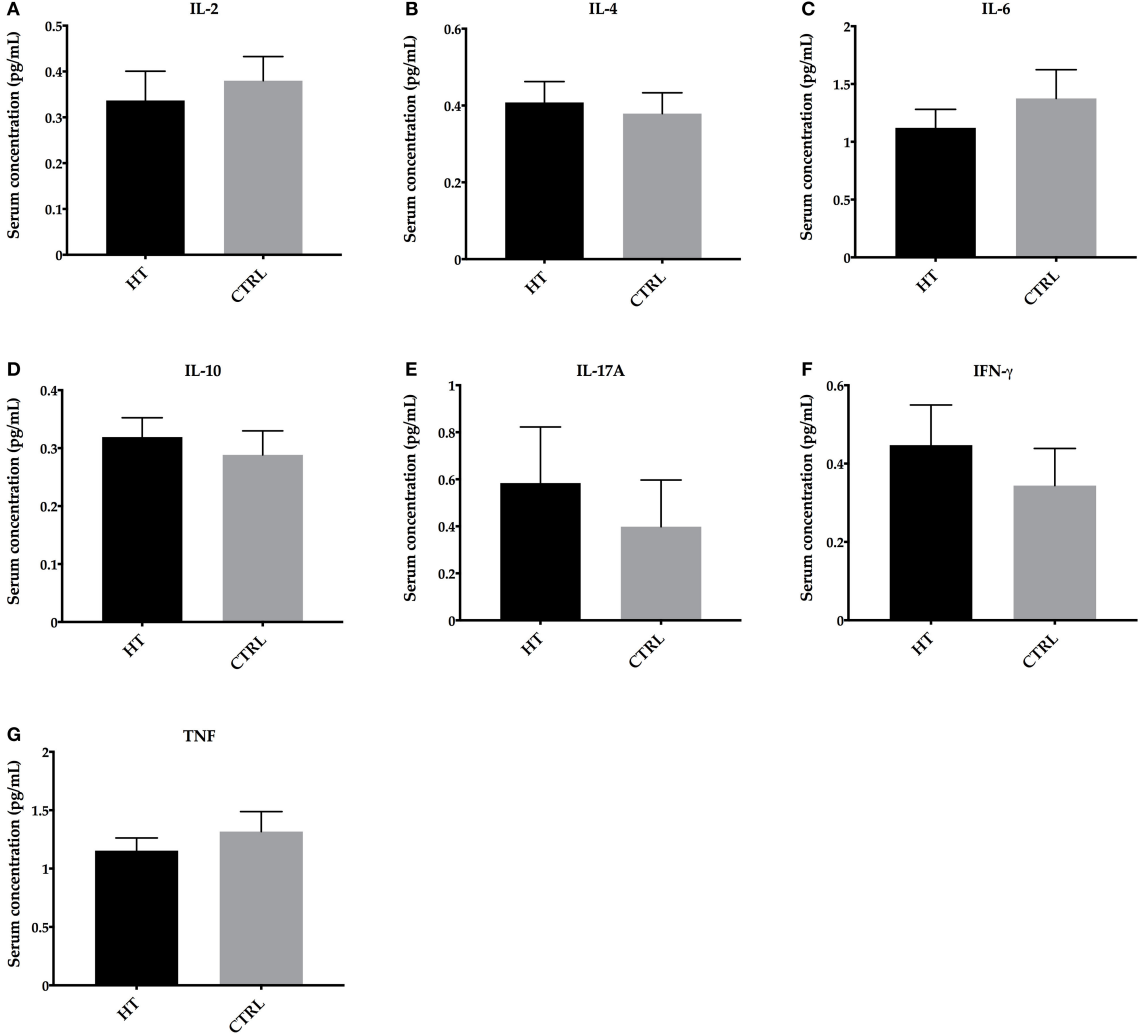
Cytokine profile in HT patients and control subjects (CTRL). Serum concentrations of **(A)** IL-2, **(B)** IL-4, **(C)** IL-6, **(D)** IL-10, **(E)** IL-17A, **(F)** IFN-γ, and **(G)** TNF. Statistical analyses were performed by Mann-Whitney. Significance was set at *P* < 0.05.

To investigate whether the alterations of intestinal microbiota impacted on systemic cytokine profile in patients with HT, we analyzed the serum concentrations of IL-2, IL-4, IL-6, IL-10, IL-17A, IFN-γ, and TNF by flow cytometer. Significant differences in these cytokines were not detected (*p* > 0.05) (mean ± SE for IL-2: 0.3369 ± 0.0638 pg/ml; IL-4: 0.4081 ± 0.0542 pg/ml; IL-6: 1.121 ± 0.1599 pg/ml; IL-10: 0.3192 ± 0.0333 pg/ml; IL-17A: 0.5835 ± 0.2388 pg/ml; IFN-γ: 0.4473 ± 0.1023 pg/ml; and TNF: 1.154 ± 0.1090 pg/ml) when compared with controls (mean ± SE for IL-2: 0.3800 ± 0.0527 pg/ml; IL-4: 0.3791 ± 0.0541 pg/ml; IL-6: 1.375 ± 0.2492 pg/ml; IL-10: 0.2884 ± 0.0414 pg/ml; IL-17A: 0.3981 ± 0.1985 pg/ml; IFN-γ: 0.3438 ± 0.0951 pg/ml; and TNF: 1.317 ± 0.1711 pg/ml) (Figures 3A–G).

### Detection of Increased Intestinal Permeability in Patients With HT

**Figure 4:**
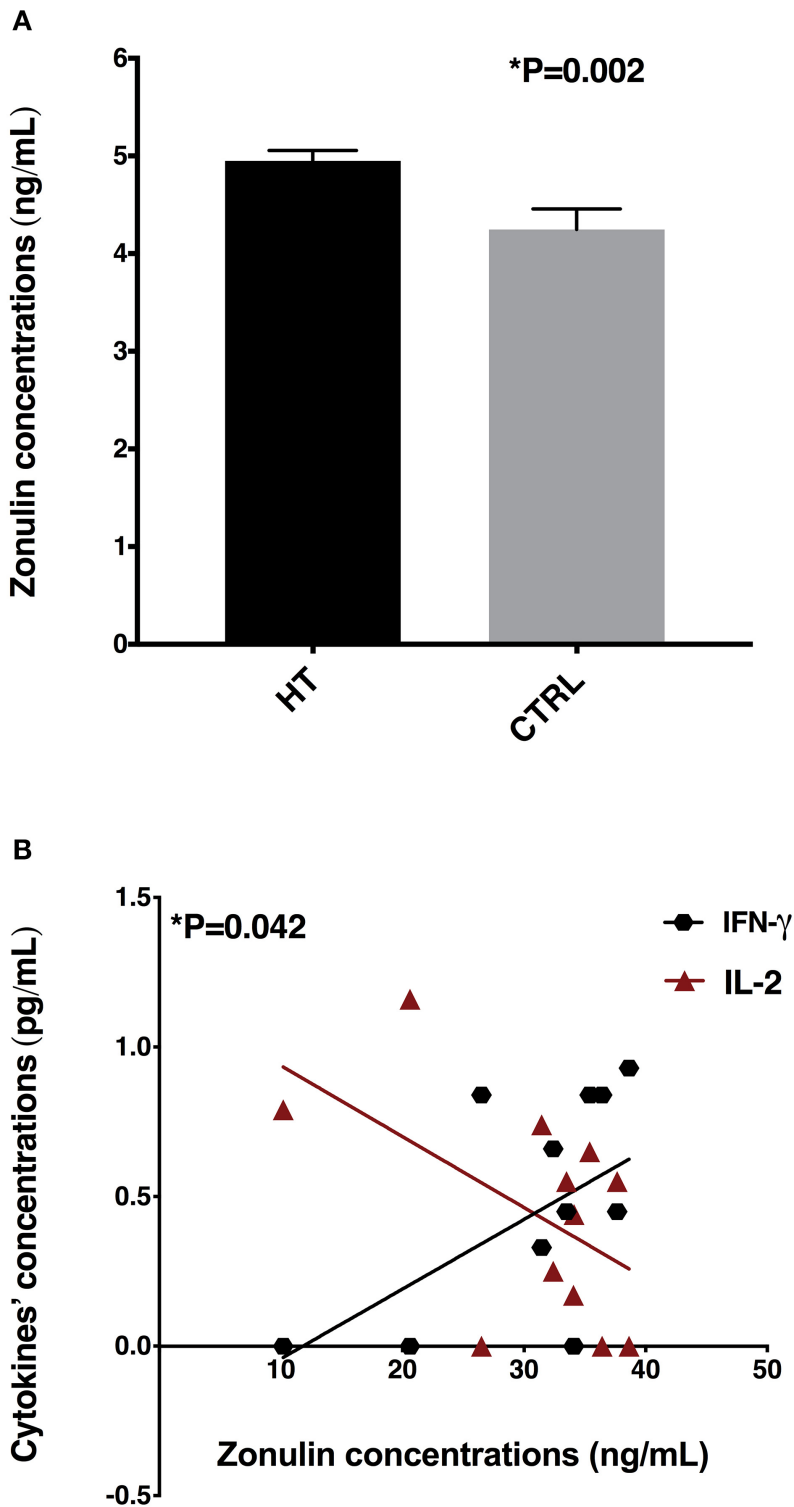
Zonulin concentrations and correlations with systemic cytokine levels. **(A)** Serum zonulin concentrations in HT patients and controls (CTRL), **(B)** Correlations among zonulin levels with IFN-γ and IL-2 concentrations.

In order to find out whether patients with HT were presented with a leaky gut, since alterations in the gut microbiota were detected, we evaluated the serum zonulin concentrations in patients with HT and controls. The zonulin serum concentrations were significantly increased (*p* = 0.002) in samples of patients with HT (mean ± SE: 30.92 ± 2.36 ng/ml) when compared with controls (mean ± SE: 19.01 ± 2.98 pg/ml) (Figure 4A). Besides that, the zonulin concentrations positively correlated with systemic IFN-γ levels (*p* = 0.042; *r* = 0.52; Figure 4B) and inversely correlated with IL-2 concentrations (*p* = 0.042; *r* = −0.51) in patients with HT (Figure 4B).

## Discussion

There is a lot of evidence that alterations in the gut microbiota are associated with autoimmune diseases development (16, 27–29). Researchers suggested that the molecular mimicry, bystander T-cell activation, post-translational modification of luminal proteins by altered microbiota, a shift to proinflammatory milieu in the gut mucosa, intestinal dysbiosis, bacterial overgrowth, and leaky gut could contribute to autoimmunity and HT trigger in genetically susceptible individuals (17, 18, 30, 31). The commensal microbiota play an important role in our physiological processes, influencing the mucosal innate and adaptive immunity and host metabolism, including carbohydrate digestion and fermentation, vitamin synthesis, hormones production and secretion, secondary bile acid synthesis, absorption of micronutrients, including iron, zinc, and iodine, which directly affect thyroid function (22, 32). In the present study, we investigated the alterations in the gut microbiota in Brazilian patients with HT and correlated these data with dietary habits, clinical data, and systemic cytokines and zonulin concentrations.

There are four studies that compared the gut microbiota from patients with euthyroid and hypothyroid HT in Chinese population, and researchers detected intestinal dysbiosis when compared with healthy controls (23, 24, 33, 34). Ishaq et al. (23) investigated the microbial composition in 29 patients with HT and 12 healthy controls by PCR-DGGE, real-time PCR, and 16S pyrosequencing (17, 19). Authors showed a reduction in Firmicutes and Bacteroidetes, and increased Proteobacteria and Cyanobacteria phyla in HT samples. Findings of the DGGE analysis showed a different fingerprint between patients and controls and the predominance of pathogenic microbes, such as *Bacteroides uniformis, B. pyogenes, B. vulgates, Shigella dysenteriae, B. intestinalis, Escherichia coli, Sporomusa ovate, Shigella flexneri*, and *Bacillus* species. By real-time PCR, authors observed a significant decrease in copy numbers of *Bifidobacterium* and *Lactobacillus*, and an increase in *E. coli* in patients with HT. By the 16S pyrosequencing, researchers detected a predominance of *Bacteroides, Escherichia–Shigella*, and *Parasutterella* genera, and a reduction in *Prevotella* and *Dialister* genera in patients with HT (23).

Another study examined the intestinal microbiota in 28 patients with euthyroid and 16 controls by 16S sequencing and showed that Firmicutes/Bacteroidetes ratio was increased and the Lachnospiraceae family was prevalent in stool of patients with HT. The abundance of *Bacteroides, Faecalibacterium, Prevotella*, and *Lachnoclostridium* genera was lower in patients with HT, and *Blautia, Ruminococcus, Roseburia, Fusicatenibacter, Romboutsia, Dorea*, and *Eubacterium* genera were higher in samples derived from patients with HT. Furthermore, the authors reported positive correlations between Firmicutes members and anti-TPO or anti-Tg autoantibodies, while Bacteroidetes members inversely correlated with these antibodies. The abundance of *Alloprevotella* positively correlated with FT4 levels, while *Romboutsia* negatively correlated with TSH concentrations (24).

In a more recent cross-sectional work, Liu et al. (33) investigated the gut microbiota in 45 patients with euthyroid, 18 patients with HT hypothyroid, and 34 controls by 16S sequencing. Compared with healthy controls, intestinal microbiota richness and diversity were significantly decreased in subjects with HT, with predominance of Bacteroidetes phylum members, *Prevotella* and *Phascolarctobacterium* species. In patients with euthyroid, some microbes, such as *Lachnospiraceae incertae sedis, Lactonifactor, Alistipes*, and *Subdoligranulum* genus, were enriched in stool samples. The authors concluded that there is a different microbiota profile in patients with diverse thyroid function, and they suggest that *Phascolarctobacterium* genus could be involved in HT progression in humans (33). *Phascolarctobacterium* species, mainly *Phascolarctobacterium faecium*, can produce acetate and propionate and influence the metabolic status of the host (35).

In accordance with the previous study, significant differences in richness and diversity of the gut microbiota were observed in 52 patients with primary hypothyroidism, compared with 40 healthy controls. The abundance of Bacteroidetes phylum members, *Veillonella* and *Paraprevotella* were significantly decreased, while *Neisseria* and *Rheinheimera* were increased in patients with hypothyroid. *Veillonella* and *Paraprevotella* genera positively correlated with FT3 and FT4 levels and were inversely associated with TSH concentrations. Researchers also showed that mRNA from enzymes involved in SCFAs production was decreased in the gut, and LPS serum levels were increased, suggesting a leaky gut in these patients. Additionally, mice that received fecal microbiota transplantation from patients with hypothyroid presented lower serum total thyroxine levels, decreased tight junction mRNA in the colon, and increased serum LPS (34).

There are neither any studies evaluating the gut microbiota in Brazilian patients with HT, nor are these data correlating with dietary habits, clinical data, and cytokines and zonulin concentrations. Here, we evaluated the gut microbiota in 40 Brazilian patients with HT and 53 healthy controls, and we observed a HT-associated microbiota imbalance, with an increase in *Bacteroides* and a significant decrease in *Bifidobacterium* genus. Our data concerning the decreased abundance of *Bifidobacterium* are in agreement with real-time PCR results from Ishaq et al. (23). *Bifidobacterium* is the first microbial colonizer of the intestines in newborns, and it plays key roles in immune system maturation and dietary metabolism (36). Some *Bifidobacterium* strains are considered probiotic because of their beneficial effects, and some studies demonstrated the beneficial role of *Bifidobacterium* species in inflammatory conditions (37, 38). The discrepancies in relation to other studies and between studies may be due to different methodologies or even due to geographic location, lifestyle, and dietary habits.

In our study, we also observed significant differences in *Lactobacillus* abundance between patients who realized thyroid hormone replacement therapy and those who did not. *Lactobacillus* are acid-lactic producing bacteria and belong to the Firmicutes phylum, the second most prevalent in the human gut (39). Recently, a study showed that *Lactobacillus* promote gut barrier integrity by producing L-Ornithine from arginine amino acid (40). We suggest that this increase in *Lactobacillus* in patients with no hormone replacement could be associated with the small intestinal bacterial overgrowth (SIBO) in patients with HT (41). Lauritano et al. studied 50 patients with HT and 40 controls and found that 54% of patients presented SIBO, in contrast with the control group (4). A clinical trial conducted by Yao et al. (42) demonstrated that the intestinal microbiota in patients with hypothyroidism, randomly divided to receive L-thyroxine (*N* = 49) or no treatment (*N* = 68), did not show significant differences with respect to alpha diversity. However, the relative abundance of *Enterococcus* and *Odoribacter* genera varied slightly depending on the dosage of L-thyroxine (42).

There are no studies correlating dietary components and gut microbiota in patients with HT. In our study, we found significant differences in dietary habits between patients with HT and controls regarding the frequency of the consumption of vegetables, fruits, animal-derived proteins, dairy products, saturated fats, and carbohydrates. Furthermore, we found an inverse correlation between animal-derived protein consumption and the abundance of *Bacteroides* genus. The main role of diet in microbiota modulation and its role in the host metabolism regulation can be exemplified by the establishment of the resident microbiota in early childhood, through the oligosaccharides ingestion from the breast milk, and also by the increase in the microbiota diversity associated with the solid food introduction (43, 44). The gut bacteria overgrowth can be directly affected by the nutrients, and these, in turn, affect both relative and absolute microbiota abundances and bacterial kinetics (45). Alterations in the ingestion of macronutrients, including proteins, carbohydrates, and fats, induce a significant shift in the gut microbiota (46). The microbiota modulation by diet directly influences on the inflammatory profile due to the generated microbiota metabolites and their direct or indirect action on immune cells from the mucosal immune system (47–51). From fermentation of dietary fibers, the intestinal microbes can generate metabolites or SCFAs with anti-inflammatory properties and preserve gut homeostasis (47). In a recent study, Sepahi et al. showed that SCFAs produced by gut microbes in response to dietary fibers influence the expansion on innate lymphoid cells in the gut through G-protein-coupled receptors and can impact the immune responses in the distant tissues depending on the host condition (51). On the other hand, dietary fats are involved in decreased microbiota diversity and richness, which increased intestinal permeability and low-grade systemic inflammation (48).

In the present study, although we demonstrated significant differences in the dietary habits and alterations in the gut microbes in patients with HT, we did not observe significant differences in the cytokine profile in patients when compared with controls. We suggest that this factor can be related to patients' diet, lifestyle, or hormone replacement and disease control. In a recent study, Tabasi et al. (52) evaluated the gut microbiota by real-time PCR and cytokine concentrations in 23 patients with obese hypothyroid and 79 obese control subjects. Researchers reported no significant differences in the gut microbes between patients with hypothyroid and controls. However, similar to our findings, the serum cytokine concentrations, including IL-1β, IL-6, IFN-γ, and TGF-β, were similar in patients and controls (52).

Finally, although there are no differences in systemic cytokines in patients with HT, we detected an increased intestinal permeability, seen by the higher zonulin concentrations in the serum of patients, when compared with controls. Similar to our study, a case–control work investigated the leaky gut in 30 patients with HT (children and adolescents), and 30 patients with congenital hypothyroidism, and reported a significant increase in serum zonulin in patients with HT. Moreover, zonulin levels positively correlated with levothyroxine dose, suggesting a connection between the increased intestinal permeability and disease severity (53). Zonulin is a physiological modulator of intercellular tight junctions, involved in macromolecules trafficking, epithelial and endothelial barrier integrity, and immune tolerance in the gut mucosa (54). Intestinal dysbiosis can activate the zonulin pathway and stimulate their release and allow the traffic of luminal contents through the epithelial barrier. The leaky gut induces inflammatory cytokines release that themselves promote an increased permeability, a vicious circle favoring the entry of antigens derived from diet and gut microbes, triggering the activation of innate and adaptive immunity in the gut mucosa (55). The main factors involved in zonulin release are the bacterial overgrowth and gluten, and the increased intestinal permeability can induce a tolerance breakdown, and then, activated immune cells can remain in the gut mucosa or migrate to distant organs, participating in chronic inflammatory and autoimmune diseases (55–57).

## Conclusions

We concluded that there are alterations in the microbiota and intestinal permeability in Brazilian patients with HT. In addition, we suggest that diet might have played an important role in modulating the gut microbiota in patients with HT. These findings could help understand the HT development and progression, while further investigations to clarify the underlying mechanisms of the diet–microbiota–immune system axis are still needed.

## Data Availability Statement

The original contributions presented in the study are included in the article/supplementary material, further inquiries can be directed to the corresponding author/s.

## Ethics Statement

The studies involving human participants were reviewed and approved by Barretos Cancer Hospital Ethics Committee (Process number 1,359/2017). The patients/participants provided their written informed consent to participate in this study.

## Author Contributions

LC and LVVS: enrollment of patients with HT, DNA extraction and quantification, cytokine determination, data acquisition, and manuscript writing. LC, GR, and AB: enrollment of controls and sample collection. AL: PCR real-time experiments and cytokine determination. LDBS: responsible for clinical data from patients with HT. JB: support for blood samples collection. EG: manuscript writing and revision. GO: experimental design, data interpretation, manuscript writing, and revision. All authors contributed to the article and approved the submitted version.

## Conflict of Interest

The authors declare that the research was conducted in the absence of any commercial or financial relationships that could be construed as a potential conflict of interest.
